# Brachiopods from the Silberberg Formation (Late Eocene to Early Oligocene) of Atzendorf, Central Germany

**DOI:** 10.1007/s12542-015-0262-8

**Published:** 2015-05-22

**Authors:** Maria Aleksandra Bitner, Arnold Müller

**Affiliations:** 1 Institute of Paleobiology, Polish Academy of Sciences, ul. Twarda 51/55, 00-818 Warsaw, Poland; 2 Institut für Geophysik und Geologie, Universität Leipzig, Talstraße 35, 04103 Leipzig, Germany

**Keywords:** Brachiopoda, *Rhynchonellopsis*, *Orthothyris*, Late Eocene, Early Oligocene, North Sea Basin, Germany, Brachiopoda, *Rhynchonellopsis*, *Orthothyris*, Obereozän, Unteroligozän, Nordseebecken, Deutschland

## Abstract

Six brachiopod species, i.e., *Discradisca* sp., *Cryptopora* sp., *Pliothyrina* sp. cf. *P. grandis* (Blumenbach, 1803), *Terebratulina tenuistriata* (Leymerie, 1846), *Rhynchonellopsis nysti* (Bosquet, 1862), and *Orthothyris pectinoides* (von Koenen, 1894), have been identified in the Late Eocene to Early Oligocene Silberberg Formation of Atzendorf, Central Germany. The species *R. nysti* and *O. pectinoides* dominate the studied assemblage. *Rhynchonellopsis* is here transferred from the family Cancellothyrididae to Chlidonophoridae because it has a loop without united crural processes. *Orthothyris pectinoides* has a brachial skeleton of chlidonophorid type, but its transverse band is incomplete. In species composition, the assemblage from Atzendorf differs from other Paleogene and Neogene European assemblages by the absence of megathyridids and dominance of chlidonophorids, indicating a relatively deep environment.

## Kurzfassung

Aus der obereozänen bis unteroligozänen Sibergerb-Formation von Atzendorf, Deutschland, konnten sechs Brachiopoden-Arten beschrieben werden: *Discradisca* sp., *Cryptopora* sp., *Pliothyrina* sp. cf. *P. grandis* (Blumenbach, 1803), *Terebratulina tenuistriata* (Leymerie, 1846), *Rhynchonellopsis nysti* (Bosquet, 1862), *Orthothyris pectinoides* (von Koenen, 1894). Die dominierenden Arten sind *R. nysti* und *O. pectinoides*. Aufgrund des Armgerüsts wird *Rhynchonellopsis* von der Familie Cancellothyrididae in die Familie Chlidonophoridae verschoben. *Orthothyris pectinoides* hat ein Brachialgerüst vom Chlidonophoriden-Typus. In Hinblick auf die Artzusammensetzung unterscheidet sich die Vergesellschaftung von Atzendorf von anderen europäischen Vergesellschaftungen durch das Fehlen von Megathyrididen und eine Dominanz von Chlidonophoriden, was auf einen relativ tiefen Ablagerungsraum hinweist.

## Introduction

Oligocene brachiopods of Europe, unlike the Eocene and Miocene ones, are still poorly known. Their presence was mentioned in several older papers (e.g., Bosquet 1862; Sandberger 1862–1863; Vincent 1893, 1923; Sacco 1902; Fabiani 1913; Venzo 1941; Meznerics 1944; Mandruzzato 1970; Nebelsick et al. 2011), but in modern taxonomy they have been so far described only from the Lower Oligocene of Germany (Bitner and Kroh 2011), and from the Upper Oligocene of Austria (Radwańska and Radwański 1989) and France (Bitner et al. 2013). Recently, a rich assemblage of Oligocene brachiopods, comprising nine genera, was briefly reported from Hungary by Dulai (2010).

The aim of this paper is to describe a brachiopod fauna from the Late Eocene to Early Oligocene Silberberg Formation at Atzendorf, Central Germany. The investigated material, very rich in specimens, allowed examination of the hitherto unknown internal structures of *Rhynchonellopsis nysti* (Bosquet, 1862) and *Orthothyris pectinoides* (von Koenen, 1894), and thus to evaluate their systematic position. Some paleoecological and paleobiogeographical aspects are also discussed.

## Geological setting

**Fig. 1:**
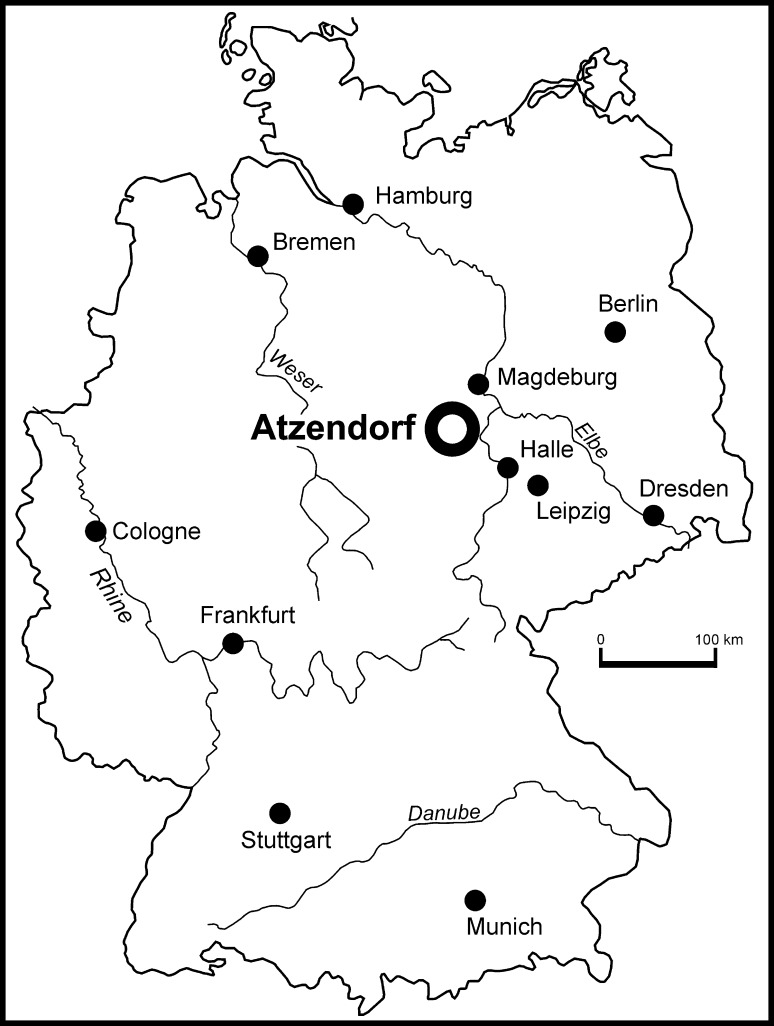
Sketch map showing the locality Atzendorf at which the specimens were collected

**Fig. 2:**
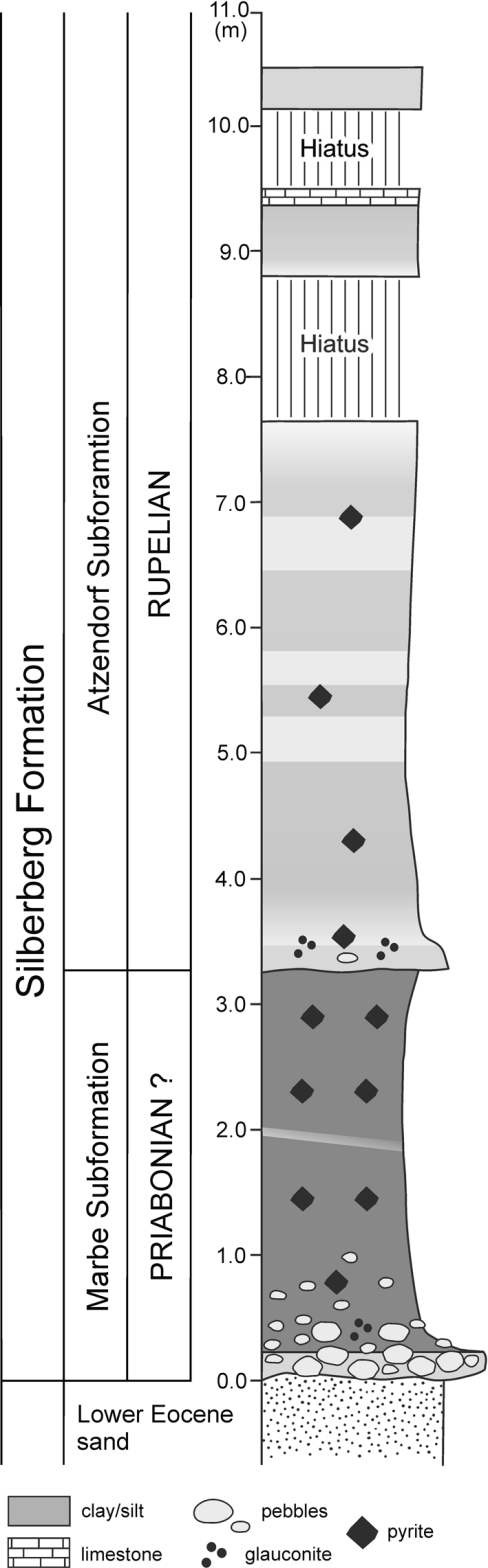
Schematic section of the Atzendorf outcrop. Modified from Müller et al. (2014)

Atzendorf is situated in Central Germany, about 100 km northwest of Leipzig (Fig. 1), and the specimens were collected from a huge, abandoned gravel pit which was recently investigated in detail by Müller et al. (2014). Paleogeographically this locality is situated on the southeastern margin of the North Sea Basin. The brachiopod-bearing deposits of the Silberberg Formation, which crop out in the Atzendorf gravel pit, lie unconformably on Lower Eocene sands and are overlain by thick Pleistocene gravels (Fig. 2). The Silberberg Formation is represented by clayey and silty deposits with a rich and diverse fauna of sponges, corals, bivalves, gastropods, brachiopods, bryozoans, echinoderms, and fishes, and is interpreted as representing a deep neritic environment (Müller et al. 2014). The formation comprises two parasequences, the lower Marbe and the upper Atzendorf Subformations. Although still under discussion, the age of this formation is considered to be uppermost Priabonian (Late Eocene) and lowermost Rupelian (Early Oligocene).

## Materials and methods

All the investigated material was collected at Atzendorf, Central Germany (Fig. 1). The gravel pit was intensively sampled between 2008 and 2010 after exploitation of the Pleistocene gravel ended. The brachiopods come from bulk samples, each of 1.5 kg dry sediment, taken each 15 cm in the seven mining dug holes, and washed in the laboratory on a 0.4-mm mesh. In addition, a few larger samples were taken at intervals of 90 cm. For details of sampling see Müller et al. (2014).

Brachiopods were found in 97 of 120 samples, which contained from 1 to 652 specimens and a total of 3103 specimens. Although most specimens are excellently preserved, there are also many damaged and/or fragmented specimens. Biodiversity indices and rarefraction were calculated using the paleontological statistics software PAST (Hammer et al. 2001).

Specimens selected for scanning electron microscopy were mounted on stubs, coated with platinum, and examined using a Philips XL-20 microscope at the Institute of Paleobiology, Warszawa. The specimens described here are housed at the University of Leipzig, Germany under catalogue numbers AZ_0686-AZ_0738.

## Systematic part

Phylum Brachiopoda Duméril, 1805

Subphylum Linguliformea Williams, Carlson, Brunton, Holmer, and Popov, 1996

Class Lingulata Gorjansky and Popov, 1985

Order Lingulida Waagen, 1885

Superfamily Discinoidea Gray, 1840

Family Discinidae Gray, 1840

Genus *Discradisca* Stenzel, 1964

*Type species Orbicula antillarum* d’Orbigny, 1845

### *Discradisca* sp.

Figure 3a–c

**Fig. 3:**
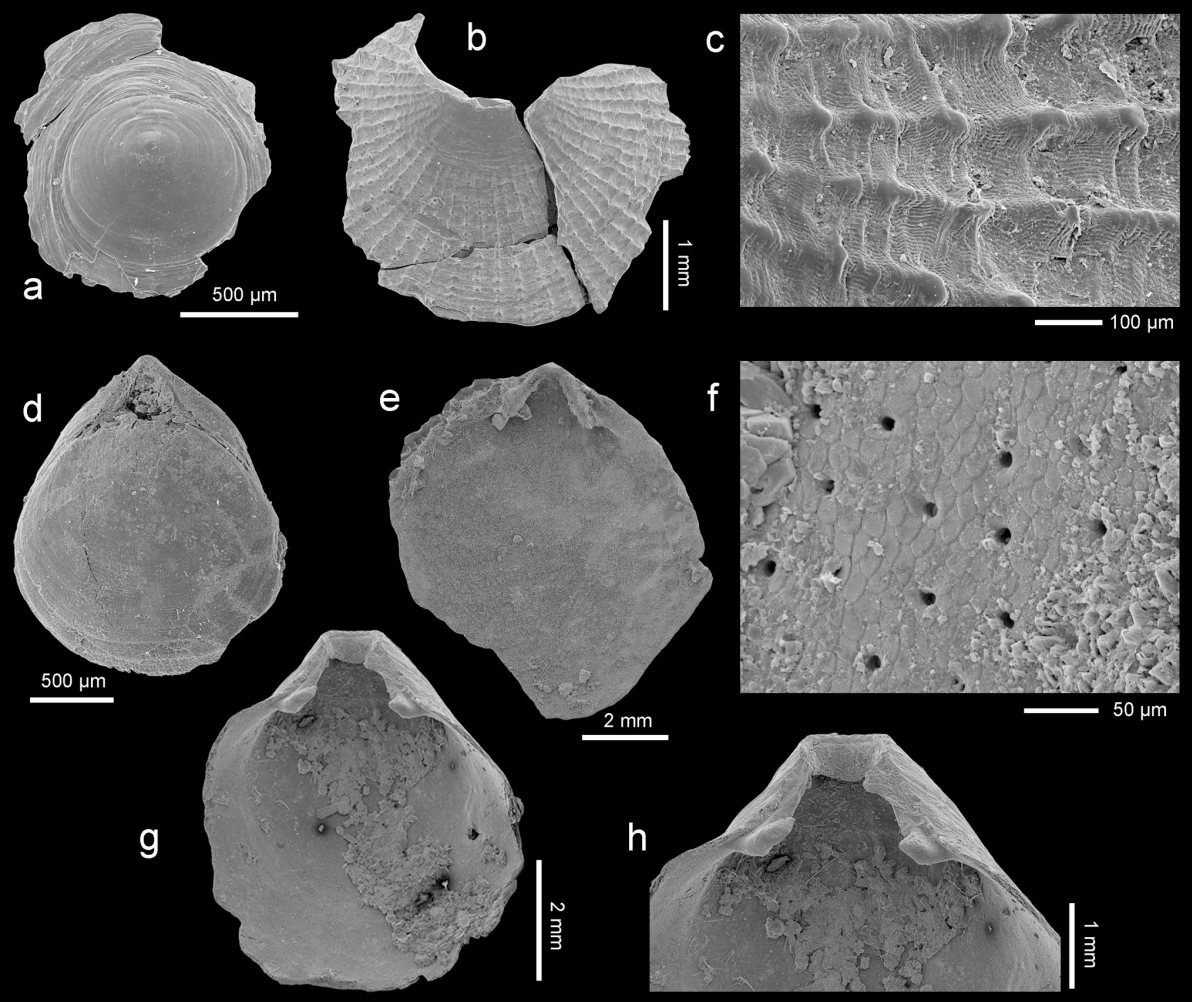
*Discradisca* sp., dorsal valves, Atzendorf, Germany: **a** outer view of postlarval (brephic) shell, broken, no. AZ_0686; **b**, **c** outer view and enlargement (**c**) of the surface to show details of microornamentation, no. AZ_0687. **d** *Cryptopora* sp., Atzendorf, dorsal view of young, complete specimen, no. AZ_0688. **e**–**h** *Pliothyrina* sp. cf. *P. grandis* (Blumenbach 1803), Atzendorf; **e**, **f** inner view of dorsal valve and enlargement (**f**) to show mosaic of secondary fibers crossed by punctae, no. AZ_0689; **g**, **h** inner view of ventral valve and enlargement (**h**) of umbonal part to show details of teeth, deltidial plates, and pedicle collar, no. AZ_0690. All SEM

*Material* Four, strongly broken, dorsal valves found in four samples.

*Remarks* The material, very limited and poorly preserved, resembles *Discinisca*, but ribbed ornamentation indicates its attribution to the related genus *Discradisca* (see discussion in Bitner and Cahuzac 2013, and Dulai 2013). The shell is thin and very small. Its postlarval (brephic) part (Fig. 3a) is smooth with numerous growth lines. The adult (neatic) shell (Fig. 3b) is covered with fine, tuberculate ribs which increase in number by intercalation. Apart from the ribs, the surface is also ornamented by numerous radial microlines (Fig. 3c). In the character of ribs and microornamentation, the studied specimens resemble *Discradisca multiradiata* (de Morgan, 1915) from the Miocene of France (Bitner and Cahuzac 2013; Dulai 2013); however, the present material prevents any conclusions.

*Occurrence* Silberberg Formation, Atzendorf, Germany. Several species of *Discradisca* have been reported from the Paleogene and Neogene deposits of Europe (see Bitner and Cahuzac 2013; Dulai 2013). Today, *Discradisca* lives in all oceans (Bitner et al. 2008; Bitner 2010, 2014).

Subphylum Rhynchonelliformea Williams, Carlson, Brunton, Holmer, and Popov, 1996

Class Rhynchonellata Williams, Carlson, Brunton, Holmer, and Popov, 1996

Order Rhynchonellida Kuhn, 1949

Superfamily Dimerelloidea Buckman, 1918

Family Cryptoporidae Muir-Wood, 1955

Genus *Cryptopora* Jeffreys, 1869

*Type species Cryptopora gnomon* Jeffreys, 1869

### *Cryptopora* sp.

Figure 3d

*Material* One complete specimen of a young individual.

*Dimensions* Length 1.9 mm, width 1.6 mm (AZ_0688).

*Remarks* The very limited material, i.e., one juvenile individual, prevents identification to species level, but it shows all characters typical of *Cryptopora*. The shell is very small, thin, and translucent, weakly biconvex with a smooth surface. The beak is high with a large, triangular, hypothyrid foramen flanked by very narrow, slightly raised deltidial plates.

The specimen was found in the lower part of the section interpreted as uppermost Priabonian (Upper Eocene), thus this is the first record of *Cryptopora* from the Eocene of Europe. From the Oligocene of Europe, a doubtful *Cryptopora* is reported from Alsace; based on description and figures, Muir-Wood (1959) attributed the specimens described as *Terebratula* (*Megerlea*) *haasi* Andreae, 1884 from the Oligocene of Lobsann to the genus *Cryptopora*.

*Occurrence* Silberberg Formation, Atzendorf, Germany. The genus *Cryptopora* is known from the Danian to the Recent, but in the Eocene and Oligocene it is very rare (Andreae 1884; Toulmin 1940; Bitner and Cahuzac 2004).

Order Terebratulida Waagen, 1883

Superfamily Terebratuloidea Gray, 1840

Family Terebratulidae Gray, 1840

Genus *Pliothyrina* van Roy, 1980

*Type species Terebratula sowerbyana* Nyst, 1843

### *Pliothyrina* sp. cf. *P. grandis* (Blumenbach, 1803)

Figure 3e–h

*Material* Two ventral valves and five dorsal valves, found in three samples; material partly damaged.

*Dimensions* Length 6.2 mm, width 5.1 mm (AZ_0690).

*Remarks* The investigated specimens are most probably juvenile representatives of *Pliothyrina grandis*, the only short-looped terebratulide reported so far from the Oligocene of Northern Europe (Davidson 1874; Vincent 1886; von Koenen 1894; Cooper 1983; Müller 2011a; Diedrich 2012). The shell is elongate oval with smooth surface and rectimarginate anterior commissure. The foramen is subcircular, mesothyrid with disjunct deltidial plates. The ventral valve has short, excavate pedicle collar and small, hooked teeth (Fig. 3h). In the dorsal valve, the cardinal process is well developed, semielliptical. The inner socket ridges are long but low; dental sockets are shallow. The loop and crura are not preserved. The shell is composed of two layers, primary and fibrous secondary (Fig. 3f); two-layered shell is characteristic for all species of *Pliothyrina* (see van Roy 1980). Although usually the shell of articulate brachiopods is built of primary and secondary layers, in the majority of short-looped, smooth terebratulides it is composed of three layers (see MacKinnon and Williams 1974; Bitner 2007, 2014).

*Occurrence* Silberberg Formation, Atzendorf, Germany. The genus *Pliothyrina* is restricted to Northern Europe, being recorded from the Oligocene to Pliocene (Cooper 1983).

Superfamily Cancellothyridoidea Thomson, 1926

Family Cancellothyrididae Thomson, 1926

Subfamily Cancellothyridinae Thomson, 1926

Genus *Terebratulina* d’Orbigny, 1847

*Type species Anomia retusa* Linnaeus, 1758

### *Terebratulina tenuistriata* (Leymerie, 1846)

Figure 4a–f

**Fig. 4:**
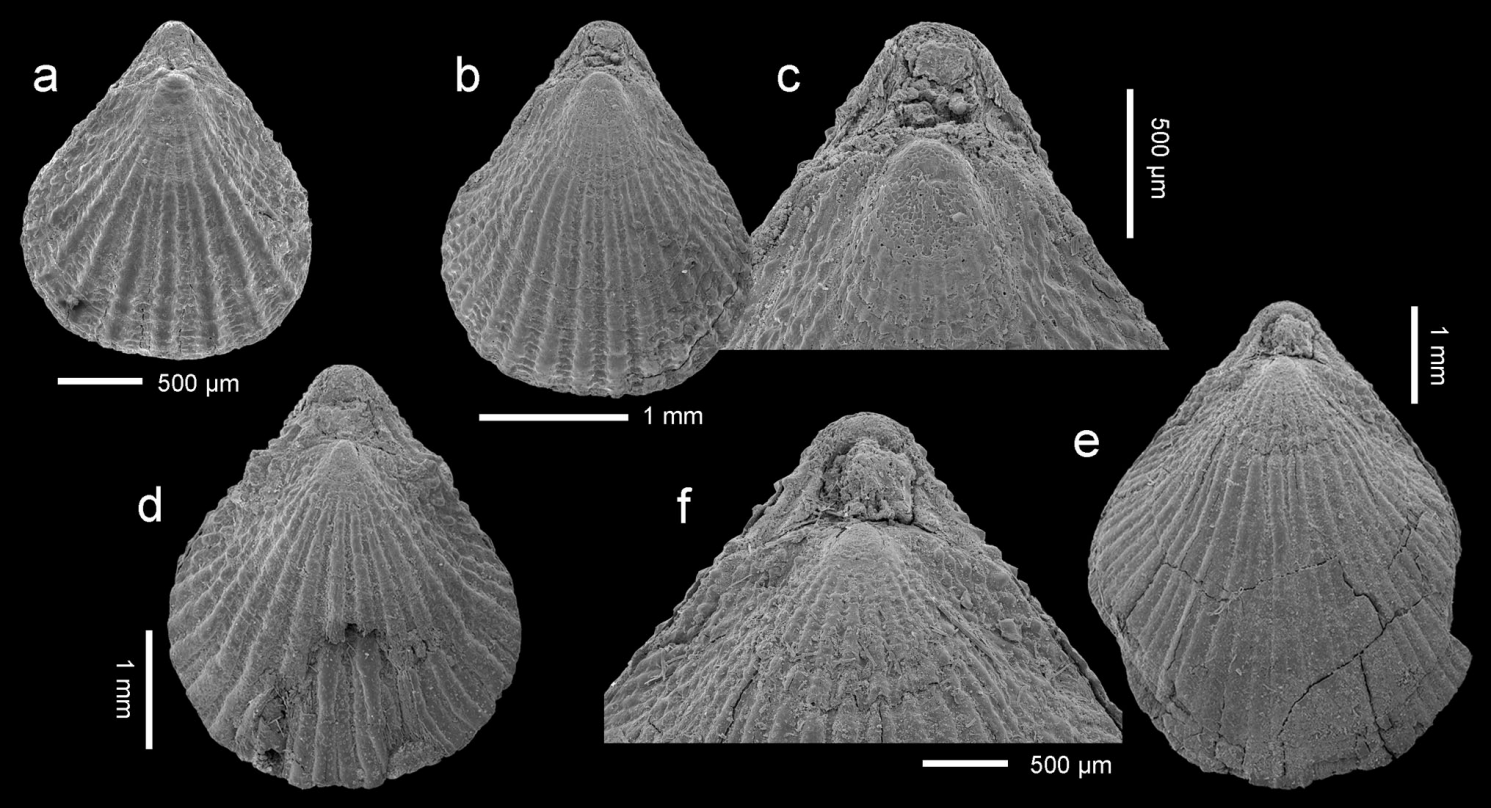
*Terebratulina tenuistriata* (Leymerie, 1846), Atzendorf, Germany: **a** dorsal view of complete specimen, no. AZ_0691; **b**, **c** dorsal view and enlargement of umbonal part of another articulated specimen (**c**) to show details of the beak, no. AZ_0692; **d** dorsal view of complete specimen, no. AZ_0693; **e**, **f** dorsal view of another articulated specimen, and enlargement of umbonal part to show details of the beak, no. AZ_0694. All SEM

2000 *Terebratulina tenuistriata* (Leymerie)—Bitner, p. 118, figs. 2, 3, 4a–f, 5a–g (*cum syn*.).

**Fig. 5:**
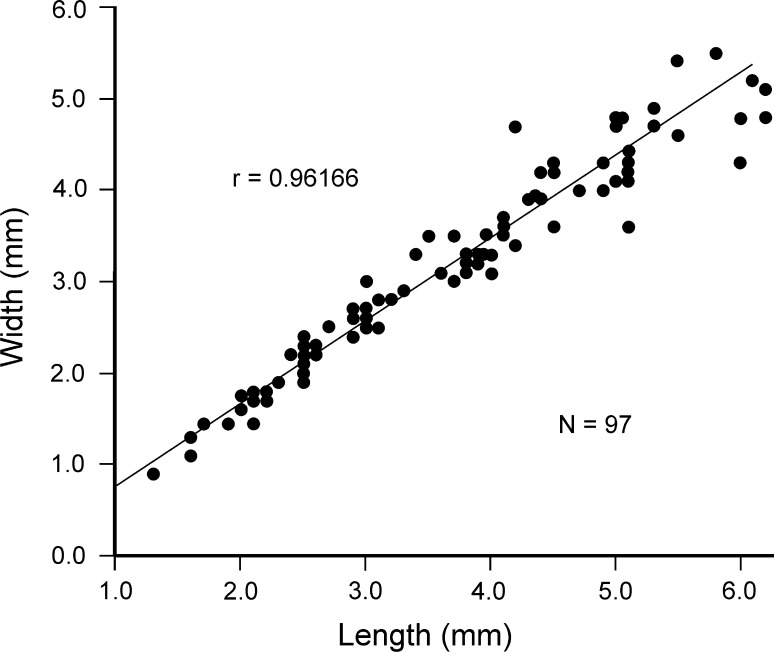
Intraspecific variation in *Rhynchonellopsis nysti* (Bosquet, 1862). Scatter diagram plotting length/width. *N* number of specimens

2005 *Terebratulina* sp. cf. *T. tenuistriata* (Leymerie)—Bitner and Dieni, p. 108, fig. 6a.

2008 *Terebratulina tenuistriata* (Leymerie)—Bitner and Dulai, pp. 33–35, fig. 4.1–8.

2009 *Terebratulina tenuistriata* (Leymerie)—Bitner and Boukhary, p. 396, fig. 3a–f.

2010 *Terebratulina tenuistriata* (Leymerie)—Dulai et al., p. 185, pl. 3, figs. 1–11.

2011 *Terebratulina tenuistriata* (Leymerie)—Dulai, pp. 299–300, fig. 4.

2011 *Terebratulina tenuistriata* (Leymerie)—Bitner et al., pp. 122–124, fig. 3a–c.

2012 *Terebratulina tenuistriata* (Leymerie)—Bitner and Boukhary, fig. 2c–d.

*Material* 83 complete specimens, 21 ventral valves, and 6 dorsal valves, found in 33 samples.

*Dimensions* (in mm)

**Table:** 

Specimen no.	Length	Width	Thickness
AZ_0694	5.0	3.9	2.1
AZ_0730	2.8	2.2	1.4
AZ_0692	2.6	2.0	1.0
AZ_0731	1.6	1.2	0.8

*Remarks* The species *Terebratulina tenuistriata* is relatively common (more than 100 specimens) in the Atzendorf assemblage. Its shell is elongate oval, biconvex, ornamented by numerous fine ribs. Although young individuals of *T. tenuistriata* and *Rhynchonellopsis nysti* are very similar, adult shells of those species are easily distinguishable. The shell in *T. tenuistriata* is much thinner and nearly equally biconvex with a large pedicle opening, while *R. nysti* has a thick shell with a strongly convex dorsal valve. Additionally, both species differ internally in loop character; in adult shells of *Terebratulina* the crural processes unite to form a ring-like loop, whereas in *Rhynchonellopsis* the crural processes are not united.

*Occurrence* Silberberg Formation, Atzendorf, Germany. In the Eocene this species has a Tethyan distribution, being common throughout Europe from Great Britain to Ukraine, but also reported from Egypt and the United Arab Emirates (see Fig. 3 in Bitner and Boukhary 2012).

Family Chlidonophoridae Muir-Wood, 1959

Subfamily Chlidonophorinae Muir-Wood, 1959

Genus *Rhynchonellopsis* Vincent, 1893

*Type species Terebratulina nysti* Bosquet, 1862

### *Rhynchonellopsis nysti* (Bousquet, 1862)

**Fig. 6:**
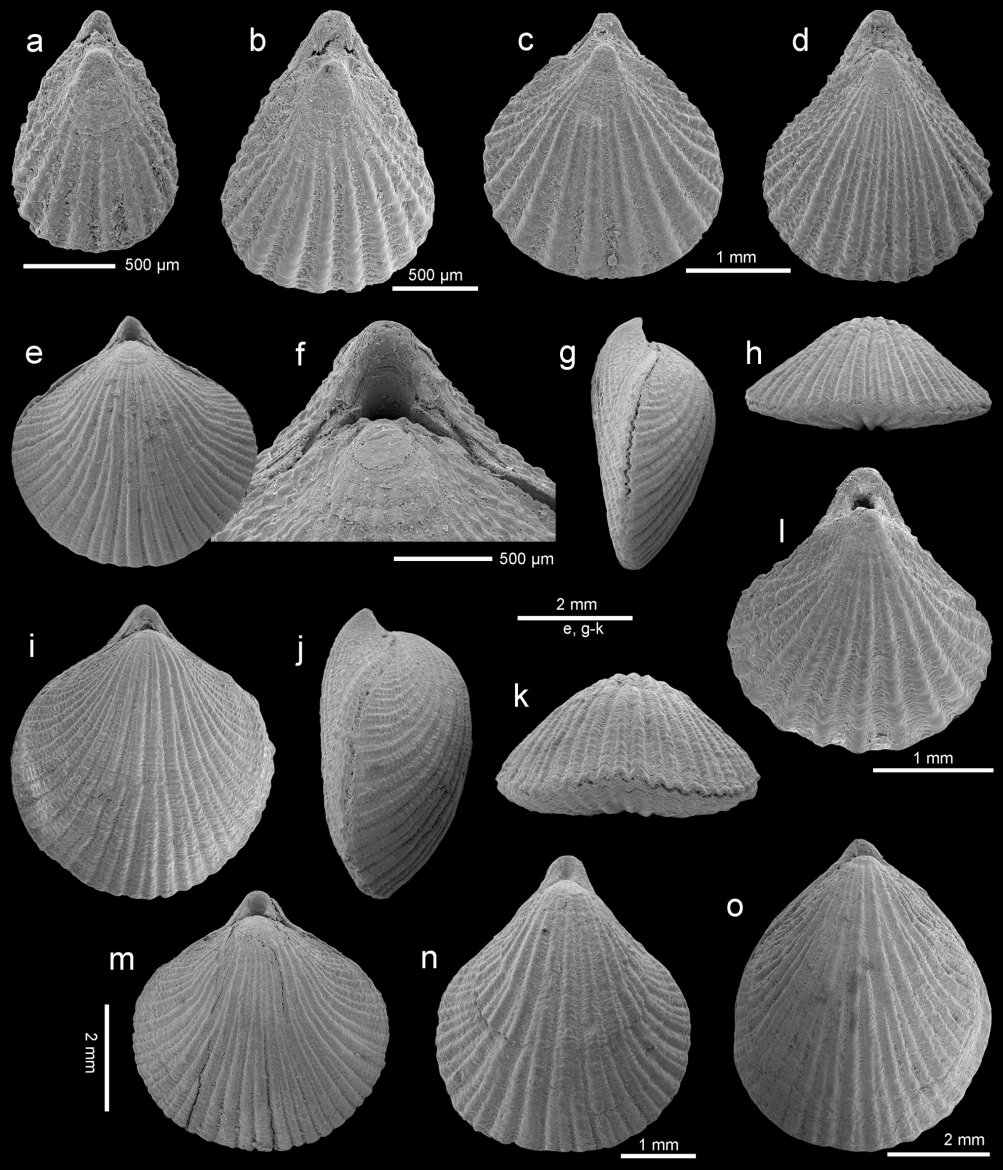
*Rhynchonellopsis nysti* (Bosquet, 1862), Atzendorf, Germany: **a**, **b** dorsal views of complete young specimens, no. AZ_0695, AZ_0696; **c**, **d** dorsal views of complete specimens, no. AZ_0697,AZ_0698; **e**–**h** dorsal, lateral, and anterior views of complete specimen, and enlargement (**f**) of umbonal part to show details of the beak, no. AZ_0699; **i**–**k** dorsal, lateral, and anterior views of complete specimen, no. AZ_0700; **l**–**o** dorsal views of complete specimens to show variability of outline, no. AZ_0701, AZ_0702, AZ_0703, AZ_0704. All SEM

**Fig. 7:**
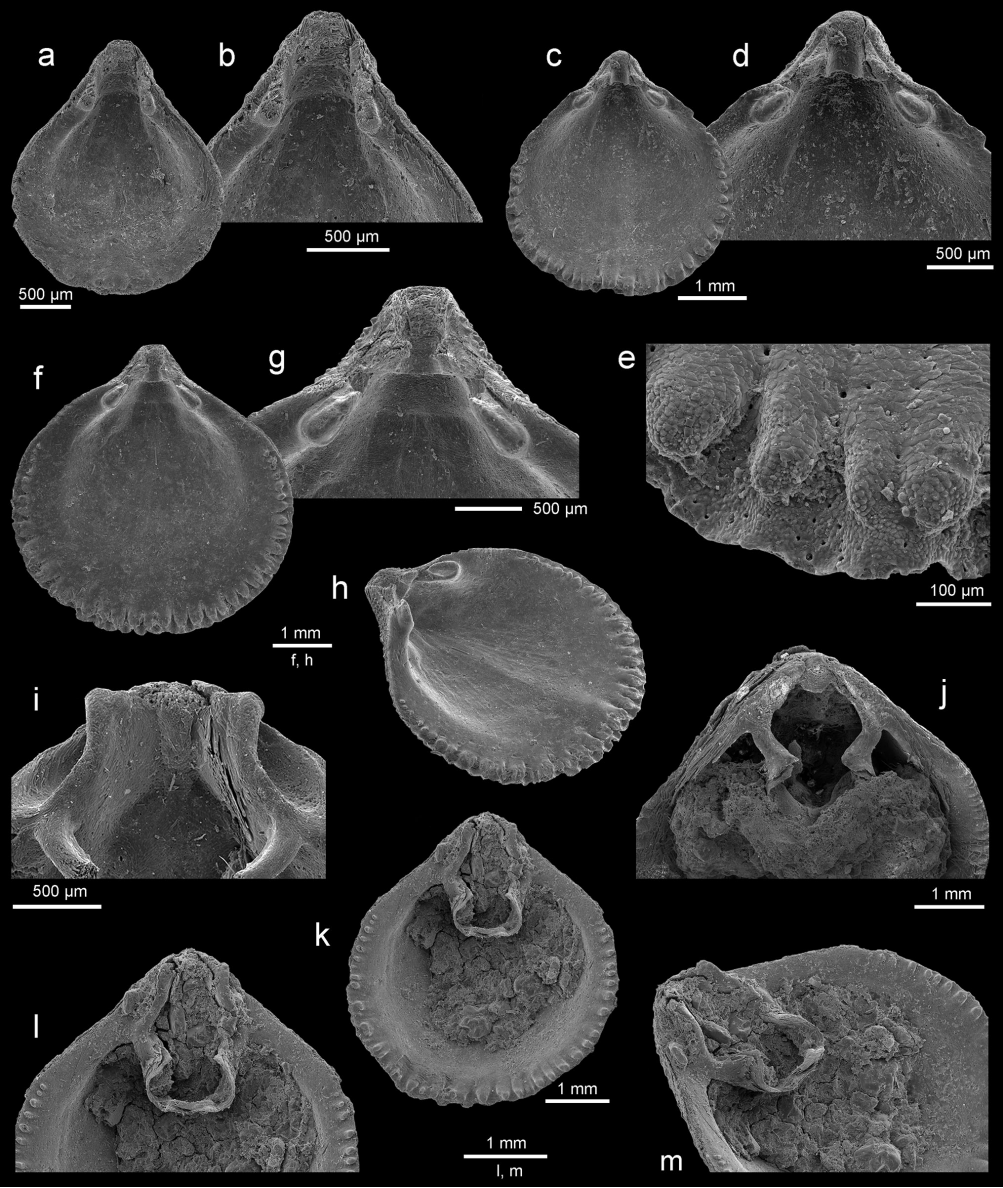
*Rhynchonellopsis nysti* (Bosquet, 1862), Atzendorf, Germany: **a**, **b** inner view of ventral valve of young individual and enlargement (**b**) of umbonal part, no. AZ_0705; **c**–**e** inner view of ventral valve and enlargement of umbonal part (**d**) and marginal tubercles (**e**), no. AZ_0706; **f**–**h** inner view of ventral valve, enlargement of umbonal part (**g**), oblique view (**h**) to show low median ridge, no. AZ_0707; **i** enlargement of umbonal part of dorsal interior to show prominent inner socket ridges and cardinal process, no. AZ_0710; **j** inner view of dorsal valve, visible loop of chlidonophorid type, no. AZ_0708; **k**–**m** inner view of dorsal valve showing complete loop, and enlargement of umbonal part (**l**) and oblique view (**m**), no. AZ_0709. All SEM

Figures 5, 6a–o, 7a–m

1862 *Terebratulina nysti* nov. spec.—Bosquet, pp. 5–6, fig. 6a, b.

1874 *Terebratulina ornata* Giebel—Davidson, p. 156, pl. 7, fig. 16.

1886 *Terebratulina ornata* Gieb.—Vincent, p. 16.

1893 *Terebratulina* (*Rhynchonellopsis*) *nysti* Bosquet—Vincent, pp. 50–52, pl. 3, figs. 12–14.

1894 *Terebratulina nysti* Bosquet—von Koenen, pp. 1352–1354, pl. 99, figs. 1–5, non fig. 6.

Non 1962 *Terebratulina nysti* Bousquet—Zelinskaya, p. 109, pl. 6, figs. 5, 6.

Non 1975 *Terebratulina nysti* Bousquet—Zelinskaya, pp. 121–122, pl. 12, figs. 10–12.

2014 *Rhynchonellopsis vincenti* (Bosquet)—Müller et al., p. 92, pl. 1, fig. 11.

*Material* 880 complete specimens, 558 ventral valves, and 420 dorsal valves, found in 85 samples.

*Dimensions* (in mm; see also Fig. 5)

**Table:** 

Specimen no.	Length	Width	Thickness
AZ_0703	6.2	4.8	2.8
AZ_0732	5.3	4.7	2.5
AZ_0699	4.4	4.1	2.1
AZ_0702	3.9	3.2	2.0
AZ_0733	2.6	2.2	1.2
AZ_0734	2.1	1.8	0.9

*Description* Shell small (maximum observed length 6.2 mm), thick, biconvex in young to strongly dorsibiconvex in adults; ventral valve nearly flat (Fig. 6g, j). Shell outline variable from rounded to elongate oval with maximum width at or nearly mid-length (Fig. 6). Shell surface covered with numerous (up to 44) beaded tuberculate ribs which increase in number by bifurcation and intercalation; in young individuals ribs are single (Fig. 6a). Lateral commissure straight, anterior commissure rectimarginate to gently uniplicate (Fig. 6h, k). Hinge line very narrow, curved. Beak short, suberect; foramen small, oval, bordered by two small, triangular, disjunct deltidial plates.

Ventral valve interior with wide pedicle collar close to the valve floor (Fig. 7b, d, g). Teeth small, hooked, with swollen bases. Very low ridge present. Dorsal valve interior with prominent inner socket ridges projecting beyond hinge line, united with cardinal process and crural bases (Fig. 7i). Hinge plates absent. Crura short but massive and converging medially (Fig. 7i, j). Crural processes blunt and short, not united. Loop short with subparallel descending branches and a horizontal to slightly inclined posterodorsally transverse band (Fig. 7j–m). Inner shell margin of both valves strongly crenulated (Fig. 7c, e, f, h).

*Remarks Rhynchonellopsis nysti* is the commonest species (more than 1800 specimens) in the assemblage under study, and its internal structure has been investigated for the first time. Originally this species was described based on a single ventral valve from Belgium (wrongly interpreted as a dorsal one), by Bosquet (1862) as *Terebratulina nysti*. Later Vincent (1893) proposed for this species a subgenus *Rhynchonellopsis*, created by himself. Having only crura preserved, Vincent (1893) offered a loop reconstruction adding a ring that became the basis for attribution of *R. nysti* to the Cancellothyrididae. However, in the Cancellothyrididae the crural processes are united and, together with a transverse band, form a ring, whereas in *Rhynchonellopsis* the crural processes are seen to be not united, as in the Chlidonophoridae (Lee et al. 2006). Thus, this genus should be transferred into the latter family.

*Rhynchonellopsis nysti* was also reported from the Upper Eocene of Ukraine by Zelinskaya (1962, 1975); however, the description clearly negates the attribution of these specimens to *R. nysti* because the dorsal valve is flat whereas in *R. nysti* the dorsal valve is strongly convex. Also based on the illustrations it is not possible to confirm the attribution to *R. nysti*; the figures can represent any cancellothyridoid (see pl. 6, figs. 5, 6 in Zelinskaya 1962, and pl. 12, figs. 10–12 in Zelinskaya 1975).

*Occurrence* Silberberg Formation, Atzendorf, Germany. Lower Oligocene of Northern Europe (Belgium, The Netherlands, Germany) (Lee et al. 2006).

Subfamily Orthothyridinae Muir-Wood, 1965

Genus *Orthothyris* Cooper, 1955

*Type species Orthothyris radiata* Cooper, 1955

### *Orthothyris pectinoides* (von Koenen, 1894)

**Fig. 8:**
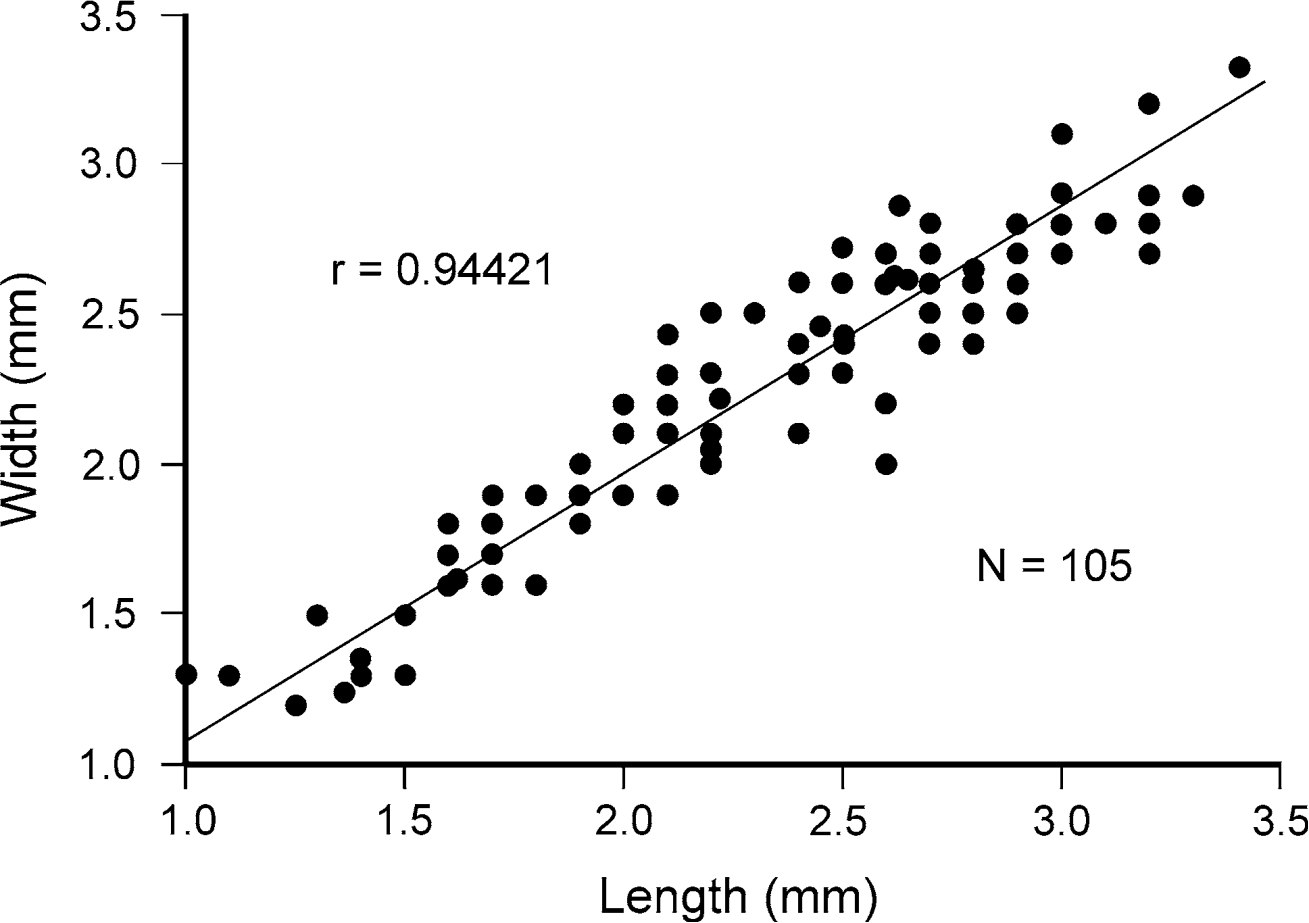
Intraspecific variation in *Orthothyris pectinoides* (von Koenen, 1894). Scatter diagram plotting length/width. *N* number of specimens

**Fig. 9:**
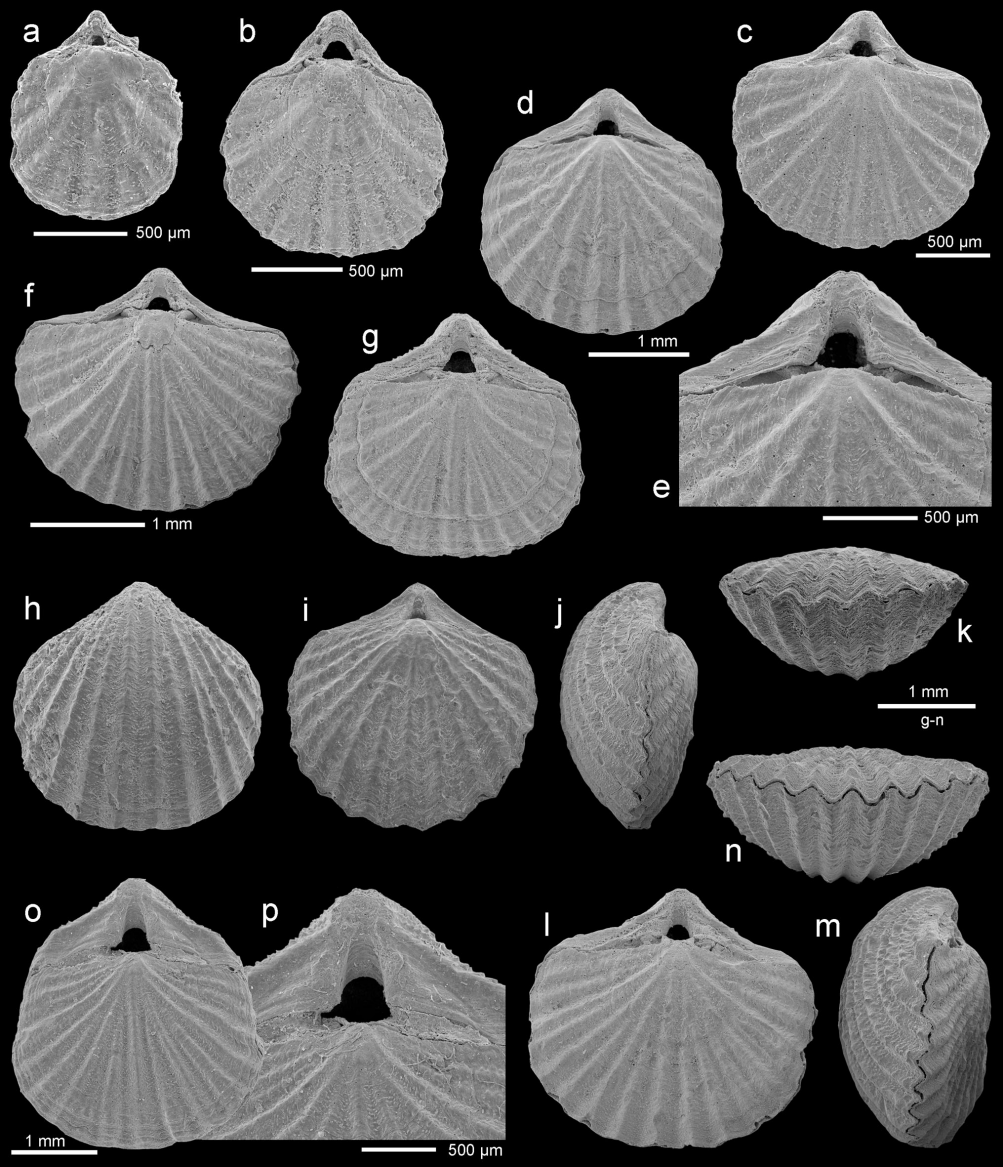
*Orthothyris pectinoides* (von Koenen, 1894), Atzendorf, Germany: **a**–**c** dorsal views of complete immature specimens, no. AZ_0711, AZ_0712, AZ_0713; **d**, **e** dorsal view of complete specimen and enlargement (**e**) of umbonal part to show details of the beak, no. AZ_0714; **f**, **g** dorsal views of complete specimens, no. AZ_0715, AZ_0716; **h**–**k** ventral, dorsal, lateral, and anterior views of complete specimen, no. AZ_0717; **l**–**n** dorsal, lateral, and anterior views of complete specimen, no. AZ_0718; **o**, **p** dorsal view of complete specimen and enlargement (**p**) of umbonal part to show details of the beak, no. AZ_0719. All SEM

**Fig. 10:**
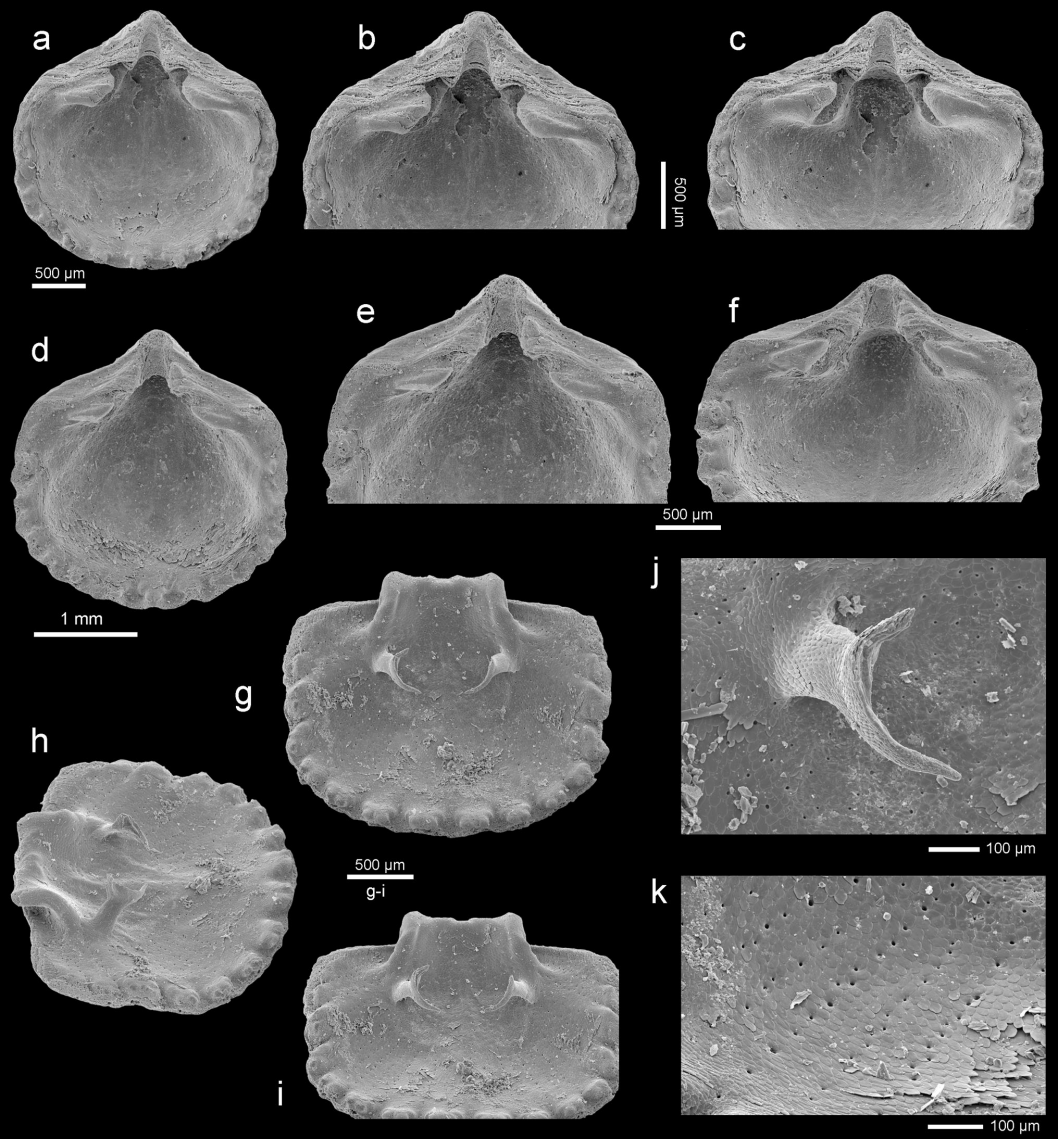
*Orthothyris pectinoides* (von Koenen, 1894), Atzendorf, Germany: **a**–**f** inner views of ventral valves, and enlargement of posterior part (**b**, **e**) and tilted views (**c**, **f**) to show deep grooves to accommodate inner socket ridges, no. AZ_0720, AZ_0721; **g**–**k** inner view of dorsal valve, oblique (**h**) and tilted (**i**) views, and enlargement of a crus with incomplete transverse band (**j**) and inner surface (**k**) to show punctae arranged in regular rows, no. AZ_0722. All SEM

Figures 8, 9a–p, 10a–k

1894 *Terebratulina pectinoides* von Koenen, pp. 1354–1355, pl. 99, figs. 8, 9.

1975 *Terebratulina pectinoides* von Koenen—Zelinskaya, pp. 116–118, pl. 13, figs. 5–19.

2005 *Orthothyris pectinoides* (von Koenen)—Bitner and Dieni, pp. 108–109, figs. 5, 6b–n.

2008 *Orthothyris pectinoides* (von Koenen)—Bitner and Dulai, p. 35, figs. 4.9–16.

2010 *Orthothyris pectinoides* (von Koenen)—Dulai et al., p. 186, pl. 2, fig. 3.

2010 *Argyrotheca sabandensis*? (Pajaud and Plaziat)—Dulai et al., p. 186, pl. 2, figs. 5–11.

2011 *Orthothyris pectinoides* (von Koenen)—Dulai, pp. 303–304, fig. 7a–n.

2011b *Orthothyris pectinoides* (von Koenen)—Müller, fig. 17.

2014 *Orthothyris pectinoides* (von Koenen)—Müller et al., p. 92, pl. 1, fig. 10.

*Material* 464 complete specimens, 199 ventral valves, and 460 dorsal valves, found in 45 samples.

*Dimensions* (in mm; see also Fig. 8)

**Table:** 

Specimen no.	Length	Width	Thickness
AZ_0719	3.2	2.9	1.8
AZ_0735	3.1	2.8	1.8
AZ_0718	2.6	2.9	1.5
AZ_0736	2.1	2.2	1.2
AZ_0737	1.7	1.9	0.8
AZ_0738	1.4	1.3	0.7

*Description* Shell small with maximum length 3.4 mm, thick, variable in outline from elongate oval, subcircular, subpentagonal to transversely oval (Fig. 9). Shell unequally biconvex with ventral valve much more convex. Shell surface ornamented by distinct, beaded ribs, up to 24 in number. Lateral commissure straight, anterior commissure incipiently, broadly sulcate. Hinge line wide, straight to slightly curved. Beak erect with well-developed interarea and strong beak ridges. Foramen small, triangular, hypothyrid; deltidial plates disjunct forming elevated ridges.

Interior of ventral valve with a well-developed pedicle collar. Teeth massive, beneath which deep grooves occur to accommodate inner socket ridges of dorsal valve (Fig. 10a–f). Dorsal valve interior with massive inner socket ridges erected beyond margin (Fig. 10g). Dental sockets deep. Posterior edge of socket ridges roughened, serving as a cardinal process. Crura short, stout, crural processes distinct. Loop short of chlidonophorid type but with an incomplete transverse band (Fig. 10g–j).

Inner commissure margin of both valves crenulated (Fig. 10d, g, h). Fibers of secondary layer readily visible on inner surface; punctae (Fig. 10k) arranged in rows associated with the rib region, a feature characteristic for Cancellothyridoidea.

*Remarks Orthothyris pectinoides* is one of the commonest species (more than 1000 specimens) in the investigated material. The specimens from Atzendorf correspond well to those hitherto described (e.g., Bitner and Dieni 2005; Bitner and Dulai 2008; Dulai 2011), being, however, larger.

The specimens from the Early Eocene of Austria described by Dulai et al. (2010) as *Argyrotheca sabandensis*? (Pajaud and Plaziat, 1972) clearly belong to *O. pectinoides*, fitting well within the variability range of this species. In addition, the specimens from Austria possess tuberculate ribs, a feature not observed in *Argyrotheca*.

*Occurrence* Silberberg Formation, Atzendorf, Germany. This species is widely distributed in the Eocene of Europe, being also reported from the United Arab Emirates (see Fig. 3 in Bitner and Boukhary 2012). In the Oligocene it is noted from Germany. Dulai (2010) mentioned *Orthothyris*? sp. from the Oligocene of Hungary.

## Discussion

**Fig. 11:**
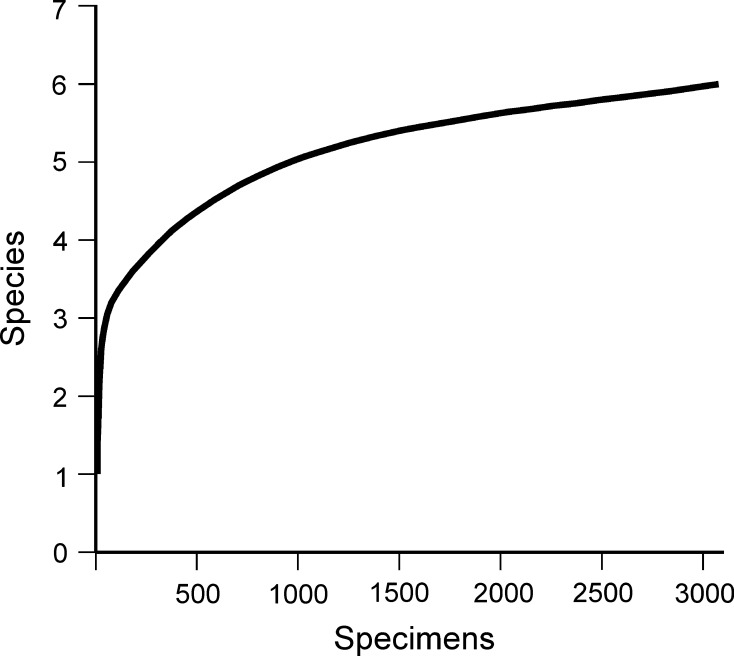
Rarefaction curve for the brachiopod sample from Atzendorf

The brachiopod fauna collected from the Late Eocene to Early Oligocene deposits at Atzendorf, Central Germany (Fig. 1) is rich in individuals but of low diversity, containing six species belonging to six genera. Nevertheless, the rarefaction curve (Fig. 11), used to test the effect of sample size upon taxon counts, begins to flatten off, suggesting that further sampling would not substantially increase the number of taxa. Diversity indices show low diversity (Shannon index = 0.82) and medium dominance (Simpson D index = 0.51).

Lingulids and rhynchonellides are represented by one species each, *Discradisca* sp. and *Cryptopora* sp., respectively. The terebratulides have four representatives: short-looped terebratuloid *Pliothyrina* sp. and three members of the superfamily Cancellothyridoidea, *Terebratulina tenuistriata*, *Rhynchonellopsis nysti*, and *Orthothyris pectinoides*. The two latter species dominate the assemblage, constituting 96 % of the material. *Rhynchonellopsis nysti* has long been considered as restricted to the Oligocene of the North Sea Basin, but the present finds extend its stratigraphical range to the Upper Eocene. However, the age of the deposits cropping out at Atzendorf is still under discussion (Müller et al. 2014). On the other hand, *T. tenuistriata* and *O. pectinoides* belong to the commonest species in the Eocene of Europe; from the Lower Oligocene they are reported from Northern Germany only.

The total absence of megathyridids (i.e., *Megathiris*, *Argyrotheca*, *Joania*), which are usually either common or dominant in other Paleogene and Neogene assemblages of Europe, makes the assemblage from Atzendorf clearly different. Today, megathyridids are commonest in shallow-water environments, preferring cryptic habitats such as overhangs, crevices, and caves (Logan 1975, 1979; Álvarez et al. 2005; Álvarez and Emig 2005), whereas the clayey deposits at Atzendorf may be interpreted as an originally soft sea bottom. The dominance of Chlidonophoridae, whose extant representatives are deep-water brachiopods (Logan 2007), indicates a deeper environment and supports previous interpretations of the Silberberg Formation (Müller 2011b; Müller et al. 2014).

All brachiopod species recognized in the Atzendorf assemblage have a functional pedicle opening, indicating that they lived attached to the hard substrate. The fauna, dominated by micromorphic species, is very similar to one described by Surlyk (1972) from the Upper Cretaceous white chalk facies. Surlyk (1972) suggested that small size may be considered as an adaptation to the availability of numerous but small hard substrates on a generally soft sea bottom. It is worth mentioning that the larger brachiopod, *Pliothyrina* sp., is rare and represented only by young individuals.

**Fig. 12:**
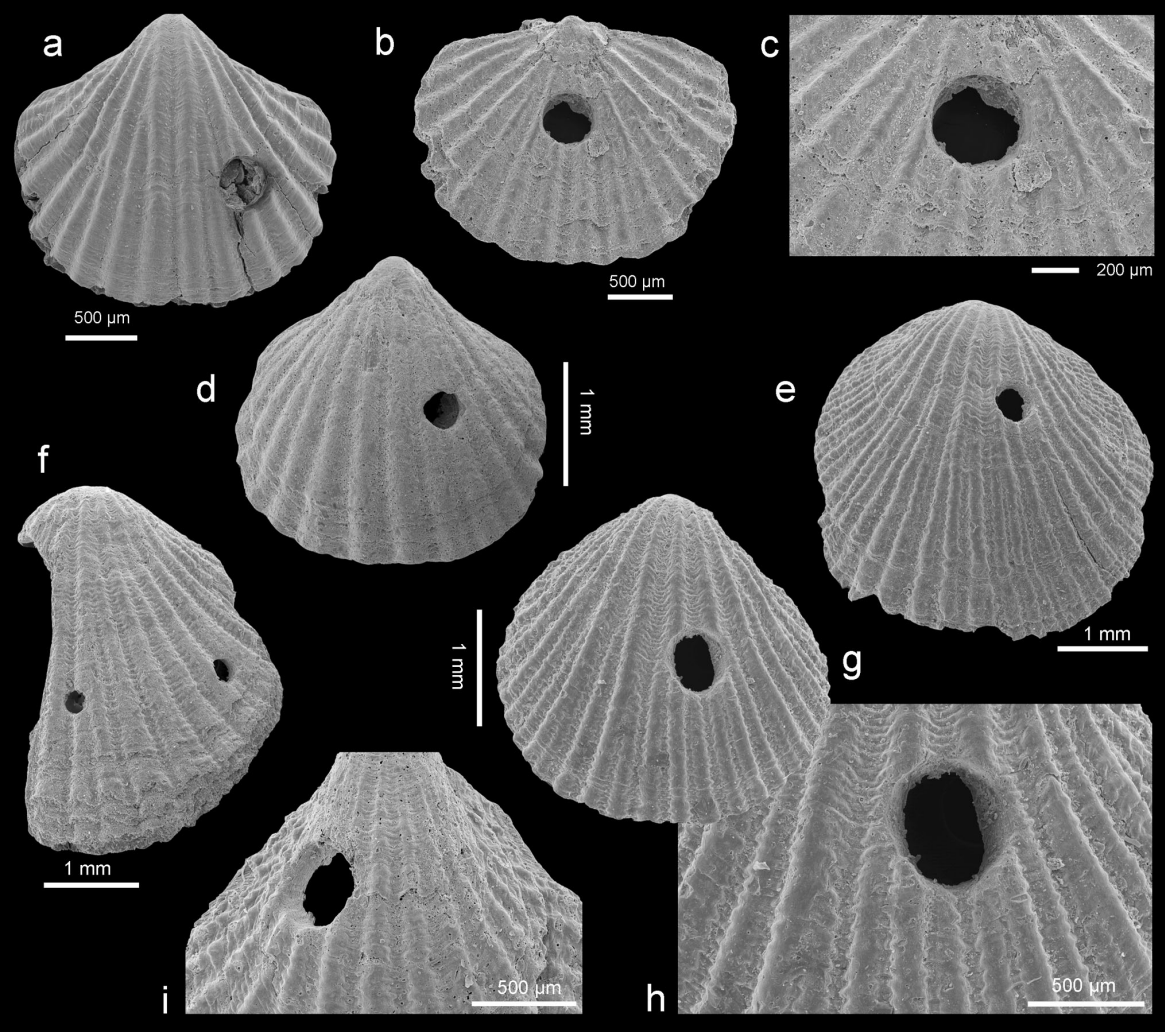
Drilled brachiopods from Atzendorf, Germany. **a**–**d** *Orthothyris pectinoides* (von Koenen, 1894), **a** ventral valve, no. AZ_0723; **b**, **c** dorsal valve and a close-up image of drill hole (**c**), no. AZ_0724; **d** ventral valve, no. AZ_0725. **e**–**i** *Rhynchonellopsis nysti* (Bosquet, 1862); **e**, **f** dorsal valves, no. AZ_0726, AZ_0727; **g**, **h** dorsal valve and a close-up image of drill hole (**h**), no. AZ_0728; **i** ventral valve, no. AZ_0729. All SEM

Traces of gastropod predation are extremely rare (Fig. 12); among 3103 specimens examined 26 (0.84 %) specimens were drilled. Such low predation intensity is a characteristic feature in Cenozoic brachiopod populations where molluscs, being more preferable food for gastropods, are abundant (compare Taddei Ruggiero and Bitner 2008; Bitner and Cahuzac 2013; Bitner et al. 2013). Drill holes have been observed only on *R. nysti* and *O. pectinoides* (see Table 1), but any apparent taxonomic selectivity can be explained by the dominance of those two species. Drill holes were found on both valves of *R. nysti* but with a preference for the dorsal valve (see Table 1). The boreholes are small (0.23–0.55 mm), rounded in outline and cylindrical (Fig. 12), corresponding to those made by extant muricids.

**Table 1:** Data on drill hole distribution in *Rhynchonellopsis nysti* and *Orthothyris pectinoides* from Atzendorf

Species	Number undrilled	Number drilled (%drilled)	Drilled on ventral	Drilled on dorsal
*Rhynchonellopsis nysti*	1837	21 (1.1 %)	4	17
*Orthothyris pectinoides*	1118	5 (0.4 %)	3	2

## Conclusions

1. The Upper Eocene to Lower Oligocene Silberberg Formation deposits at Atzendorf, Central Germany yielded an abundant brachiopod fauna of low diversity. The assemblage consists of six species, *Discradisca* sp., *Cryptopora* sp., *Pliothyrina* sp. cf. *P. grandis*, *Terebratulina tenuistriata*, *R. nysti*, and *O. pectinoides*. The two latter species dominate.
2. The internal structures of *R. nysti* and *O. pectinoides* were investigated for the first time. *Rhynchonellopsis nysti* possesses a loop with disjunct crural processes and therefore has been transferred from the family Cancellothyrididae to the family Chlidonophoridae. *Orthothyris pectinoides* also has a loop of chlidonophorid type with an incomplete transverse band.
3. The dominance of chlidonophorid brachiopods and absence of megathyridids indicate a deeper-water environment.
4. Gastropod predation intensity on brachiopods is very low; drillings were observed on fewer than 1 % of specimens.
